# Characterization of Serotype CD Mosaic Botulinum Neurotoxin in Comparison with Serotype C and A

**DOI:** 10.3390/toxins15020123

**Published:** 2023-02-03

**Authors:** Shin-Ichiro Miyashita, Shura Karatsu, Mako Fujiishi, I Hsun Huang, Yuki Nagashima, Tamaki Morobishi, Keita Hosoya, Tsuyoshi Hata, Min Dong, Yoshimasa Sagane

**Affiliations:** 1 Department of Food, Aroma and Cosmetic Chemistry, Faculty of Bioindustry, Tokyo University of Agriculture, Abashiri 099-2493, Hokkaido, Japan; 2 Department of Urology, Boston Children’s Hospital, Boston, MA 02115, USA; 3 Department of Microbiology, Harvard Medical School, Boston, MA 02115, USA; 4 Department of Surgery, Harvard Medical School, Boston, MA 02115, USA

**Keywords:** botulinum neurotoxin, mosaic toxin, serotype CD, muscle paralysis, botulism, *Clostridium botulinum*, half-life

## Abstract

Botulinum neurotoxin (BoNT), produced by *Clostridium botulinum*, cleaves proteins involved in neurotransmitter release, thereby triggering flaccid paralyses, which are responsible for botulism. BoNT is classified into seven serotypes (BoNT/A-G); BoNT/A and BoNT/B are used as medical therapeutics and anti-wrinkle reagents. In this study, we investigated the efficacy of BoNT/CD, a mosaic toxin of BoNT/C and BoNT/D, to assess its potential as a therapeutic alternative for BoNT/A. In a cultured neuron assay, BoNT/CD cleaved syntaxin and SNAP-25 with higher efficacy than BoNT/C and BoNT/A. Intramuscularly administrated BoNT/CD induced dose-dependent muscle paralysis, and the paralysis lasted ~21 days in a mouse digit abduction score assay (BoNT/A-induced paralysis lasted ~30 days). BoNT/C failed to induce local paralysis without systemic toxicity. Multiple alignment analyses of the amino acid sequences of the receptor binding domain (H_C_) of eight BoNT/CDs and two BoNT/Ds showed sequence clustering in five groups. Comparing BoNT/CD strain 003-9 (BoNT/CD_003-9_) and strain 6813 (BoNT/CD_6813_) showed that both BoNT/CDs displayed similar efficacies in cultured neurons, but BoNT/CD_003-9_ displayed higher efficacy in a mouse model than BoNT/CD_6813_. These findings suggest that BoNT/CD may be a potential alternative for patients who do not respond to existing BoNT-based therapeutics.

## 1. Introduction

Botulinum neurotoxin (BoNT), the most potent toxin in nature, is produced by *Clostridium botulinum*. The BoNT induces flaccid paralyses in humans and animals, and this condition is known as botulism. BoNT is classified into seven serotypes based on their antigenicity (BoNT/A-G). BoNT/A, /B, and /E (rarely BoNT/F) dominantly cause human botulism, and BoNT/C and BoNT/D induce botulism in animals [1]. Due to its extreme toxicity, BoNT is considered a potential biological warfare agent [2]. In contrast, BoNT has been used by clinicians to treat muscle disorders, and it was approved by the United States Food and Drug Administration as a therapeutic in the late 1970s [3,4,5]. BoNT/A, in particular, is the most widely used because it induces long-term muscle paralysis due to its long half-life in the motor neurons [6]. Once the BoNT-induced paralysis subsides, the patient needs to be reinjected with the toxin. However, this process may be negated by the immune response and may generate the same serotype of the BoNT for the secondary non-responder patient [7]. Thus, it would be useful to explore if other BoNT serotypes can be used as alternatives to BoNT/A.

The BoNT is produced as a single-chain polypeptide (150 kDa), and it is thereafter post-translationally cleaved by bacterial or exogenous protease(s), yielding the active form consisting of two chains—a light chain (LC, catalytic domain, 50 kDa) and heavy chain (HC, 100 kDa)—connected by a disulfide bond. The HC can be further divided into two functional domains: the translocation domain (H_N_) and the receptor-binding domain (H_C_), 50 kDa each. The H_C_ domain binds to the post-synaptic terminals in the neuromuscular junction with extraordinarily high affinity. The BoNT binds to the cell membrane and enters the cell via receptor-mediated endocytosis, and the acidification in the endosome induces a conformational change in H_N_. The H_N_ mediates the transport of the LC into the cytosol. The LC, which is a metalloprotease, cleaves the SNARE proteins, which are necessary for the release of neurotransmitters from synaptic vesicles to the post-transmembrane in the neuron. The inhibition of the release of these transmitters causes flaccid muscle paralysis [4,8,9].

The muscle paralysis caused by BoNTs can vary in time ranges from weeks to several months: BoNT/A (~9 months), BoNT/C (4-6 months), BoNT/B (2-4 months), BoNT/D (~3 weeks), and BoNT/E (2–3 weeks) [6,10,11]. The reason why BoNT/A has a significant long-lasting effect remains unclear, but it has been reported that interactions with septin [12] and deubiquitinase [13] are involved. In addition to their long-lasting effect, BoNT/A and BoNT/B can induce muscle paralysis at very specific sites when the toxin is injected into the muscle, and they are often utilized in muscle disorders for medical and cosmetic purposes. BoNT/C also induces muscle paralysis with relatively long-lasting effects [14,15,16]. However, it is thus far unknown whether BoNT/C can induce local paralysis without the diffusion of the toxins. Meanwhile, BoNT/D binds to synaptic vesicle protein 2 (SV2) proteins and gangliosides in presynaptic terminals in the neuromuscular junction, similarly to BoNT/A [17,18]. 

To improve the efficacy of the BoNT, chimeric toxins have been artificially produced as recombinant proteins [19,20]. In these reports, the potency of BoNT was improved by the replacement of the functional domains in the BoNT molecule with other serotypes to achieve longer-duration paralysis or change its sensitivity to the targeted neurons. In contrast, there are natural mosaic BoNT molecules consisting of functional domains from different serotypes generated by horizontal gene shuffling between the distinct serotypes of BoNT genes (BoNT/FA [21], BoNT/DC, and BoNT/CD [22,23,24]). 

**Figure 1:**
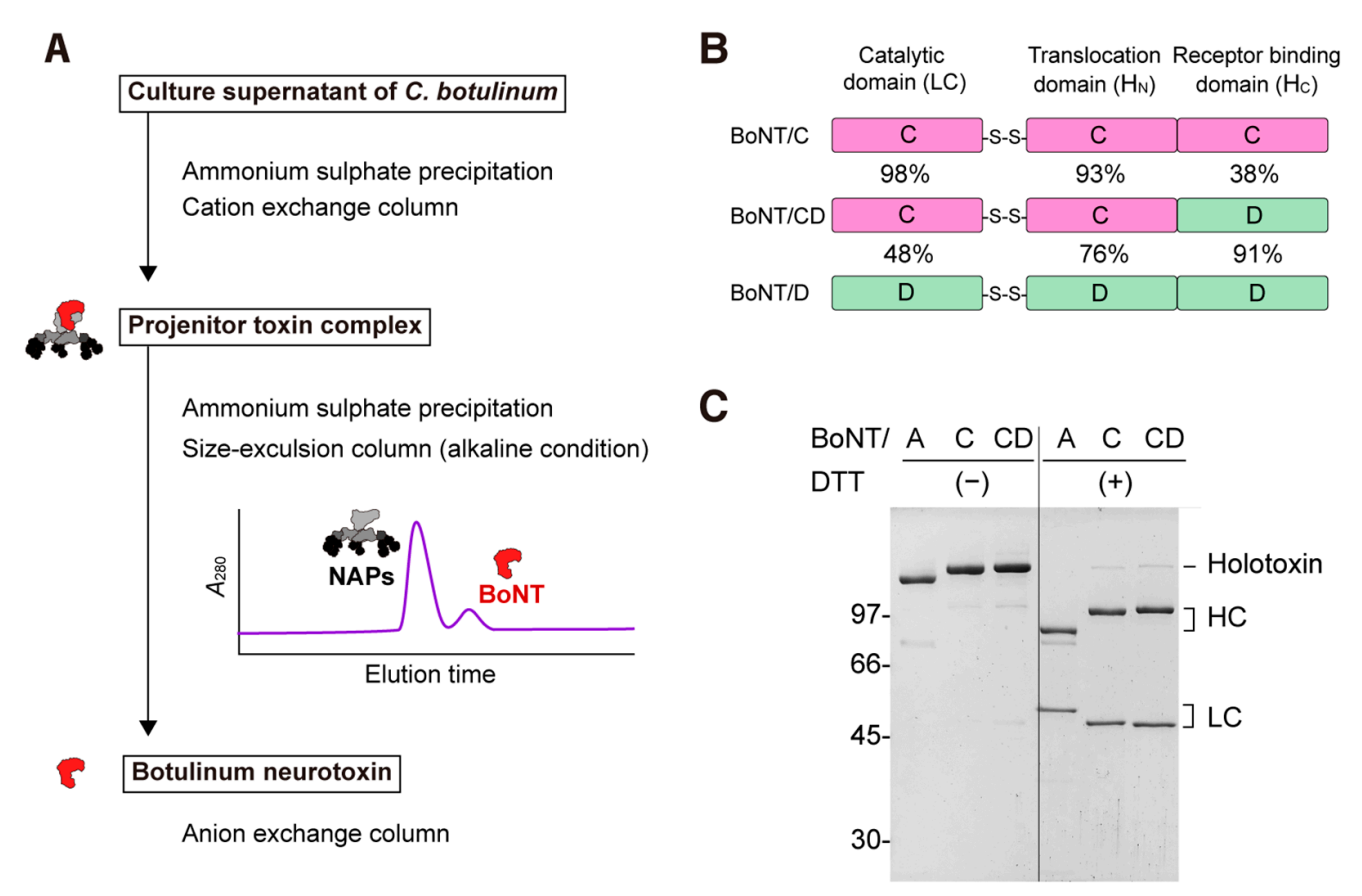
Production and characterization of BoNT/CD_003-9_. (**A**) The flowchart outlines the purification of botulinum neurotoxins (BoNTs). BoNTs were dissociated from the progenitor-toxin complex by dialysis under a pH of 8.8 and separated into neurotoxin-associated proteins (NAPs) by the size-exclusion column. (**B**) Schematic illustration of the BoNT/C strain Stockholm, BoNT/CD_003-9_, and BoNT/D strain CB16. The percentages indicate the sequence identity of each domain between BoNT/CD_003-9_, BoNT/C, and BoNT/D. (**C**) Purified BoNTs from the culture supernatant of *C. botulinum*. BoNTs (1.5 µg) were analyzed on SDS-PAGE without or with DTT.

In this study, we characterized BoNT/CD in a local muscle paralysis mouse model. The BoNT/CD is one of the natural mosaic toxins comprising serotypes C LC and H_N_ (LC-H_N_/C) and D H_C_ (H_C_/D) (see Figure 1). BoNT/D has been tested in humans and resulted in poor effectiveness, although BoNT/D utilizes SV2 as a protein receptor, which is also a receptor for BoNT/A [25,26]. Meanwhile, BoNT/C may utilize only gangliosides, with no protein receptors known [27,28,29,30,31]. BoNT/C has been considered as an alternative to BoNT/A because the LC/C demonstrates long-lasting neuroparalysis both in cultured rodent neurons and in humans [14,32,33]. Here, we observed that BoNT/CD induced local and long-lasting paralysis to a similar degree to that of BoNT/A and is superior to BoNT/C. Thus, BoNT/CD may be an alternative candidate to BoNT/A or BoNT/B for medical and cosmetic use.

## 2. Results

### 2.1. Domain Architecture and Purification of BoNT/CD

To further explore the alternatives to BoNTs, we characterized the mosaic neurotoxin BoNT/CD. The BoNT exits as a progenitor toxin complex (PTC) in which the BoNT is associated with neurotoxin-associated non-toxic proteins (NAPs) in the acidic to neutral pH range [34,35]. The PTCs of BoNT/CD (BoNT/CD_003-9_; strain 003-9), BoNT/C (BoNT/C_YOI_; strain Yoichi, for which its amino acid sequence is identical to that of the Stockholm strain), and BoNT/A (strain 62A) were purified from the culture supernatant of their native *C. botulinum* strains using ammonium sulfate precipitation and ion-chromatography. To isolate BoNTs (150 kDa), the PTCs (750 kDa) were brought to alkaline conditions (pH 8.8) and placed in the size-exclusion and anion-exchange columns (Figure 1A).

The amino acid sequence of BoNT from the serotype CD strain 003-9 employed in this study was compared with those of typical BoNT/C (serotype C strain Stockholm; BoNT/C_STO_) and BoNT/D (serotype D strain CB16) to characterize the domain architecture of the toxin, as shown in Figure 1B. The LC and H_N_ amino acid sequences of the BoNT/CD_003-9_ showed 98% and 93% similarity with those of BoNT/C_STO_, respectively. On the other hand, the H_C_ of BoNT/CD_003-9_ shares 91% amino acid sequence identity with H_C_/D, but it only shares 38% identity with H_C_/C. These data indicate that the BoNT/CD_003-9_ is a mosaic BoNT/CD protein consisting of a BoNT/C LC and H_N_ and BoNT/D H_C_ [23]. 

As a result, the BoNT/A, BoNT/C_YOI_, and BoNT/CD_003-9_ were purified to ~95% purity (Figure 1C). SDS-PAGE analyses demonstrated that these BoNTs gave a single band in the absence of reducing agents. In the presence of dithiothreitol, the BoNTs were separated into two fragments of LC (50 kDa) and HC (100 kDa). These data indicated that the purified BoNTs were already activated by protease(s) during the *C. botulinum* culture. Thus, the activation of the BoNT by exogenous protease is not needed [36].

### 2.2. BoNT/CD_003-9_ More Efficiently Cleaved SNARE Proteins than BoNT/C and BoNT/A in Rat-Cultured Cortical Neurons

**Figure 2:**
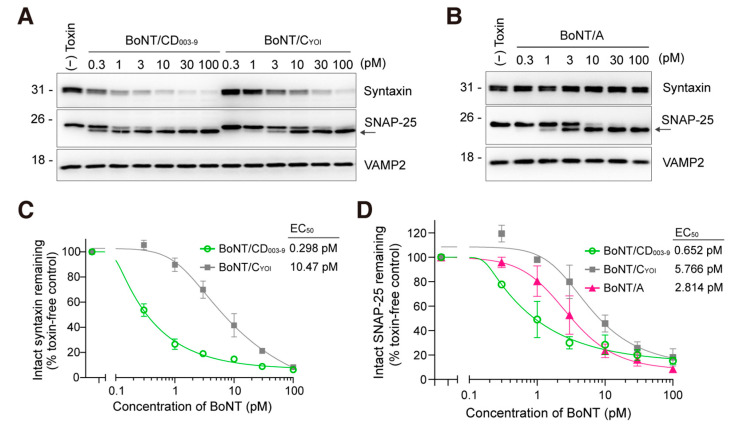
BoNT/CD_003-9_ is more potent than BoNT/C_YOI_ or BoNT/A in cultured neurons. (**A**,**B**) Representative Western blot images of the SNARE protein cleavage by BoNT in cultured rat cortical neurons. Neurons were exposed to the indicated concentrations of BoNT/CD_003-9_, BoNT/C_YOI_ (panel (**A**)), or BoNT/A (panel (**B**)) for 12 h. The cleavage of SNARE proteins in neuron lysate was analyzed by Western blotting. The arrow indicates the cleaved SNAP-25. VAMP2 served as a loading control. (**C**,**D**) Quantitative analysis of SNARE protein cleavage by BoNT. The band intensities were analyzed by ImageJ software. Intact syntaxin or SNAP-25 was plotted against BoNT-untreated cells (panels (**C**,**D**)). The data are means ± S.E. of three independent biological replicates.

We then compared the efficacy of BoNT/CD_003-9_, BoNT/C_YOI_, and BoNT/A in rat-cultured cortical neurons. Neurons were exposed to a pico-molar level of BoNT for 12 h. BoNT/CD_003-9_ and BoNT/C_YOI_ cleaved syntaxin and SNAP-25 with different efficacies (Figure 2A). BoNT/A cleaved SNAP-25 (Figure 2B). The cleavage of syntaxin by BoNT/CD_003-9_ was thirty-five-fold higher than that by BoNT/C, with EC_50_s of 0.298 pM and 10.47 pM, respectively (Figure 2C). The EC_50_ values for SNAP-25 cleavage by each BoNT were 0.652 pM (BoNT/CD_003-9_), 5.766 pM (BoNT/C_YOI_), and 2.814 pM (BoNT/A) (Figure 2D). These results indicate that BoNT/CD_003-9_ displays the highest efficacy in cultured rat cortical neurons among the BoNTs tested.

### 2.3. Local Muscle Paralysis by BoNT/CD in Mice

**Figure 3:**
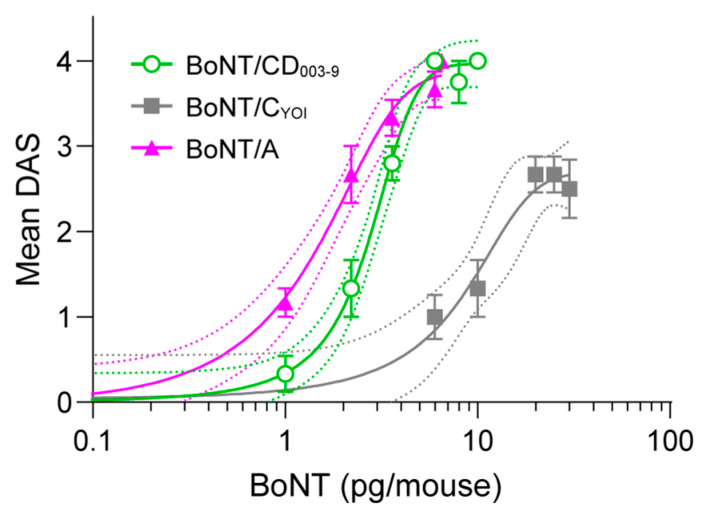
Dose-response of BoNT in the DAS assay. BoNT was intramuscularly injected into the mouse’s hind leg muscle, and muscle paralysis was monitored. The means of DAS scores at 3 days post-BoNT injection were scored and plotted. The data are means ± S.E. of six animals per dose.

To investigate whether BoNT/CD_003-9_ can induce local muscle paralysis, we next performed the mice digit abduction score (DAS) assay, which is a non-lethal method for measuring local muscle paralysis [37]. Figure 3 shows the DAS scores at 3 days post-injection of BoNT/CD_003-9_, BoNT/A, and BoNT/C_YOI_. At 3 days post-injection, both mice injected with 6 pg of BoNT/A and BoNT/CD_003-9_ showed similar DAS scores of 4. The mice injected with 1 pg of BoNT/A displayed a relatively higher DAS score (1–2) than those injected with 1 pg of BoNT/CD_003-9_ (0–1). On the other hand, mice injected with 6–30 pg of BoNT/C_YOI_ displayed a DAS score of only 1–3 and did not reach the maximum of 4. This result indicates that the efficacy of these BoNTs, in descending order, is BoNT/A, BoNT/CD_003-9_, and BoNT/C_YOI_.

**Figure 4:**
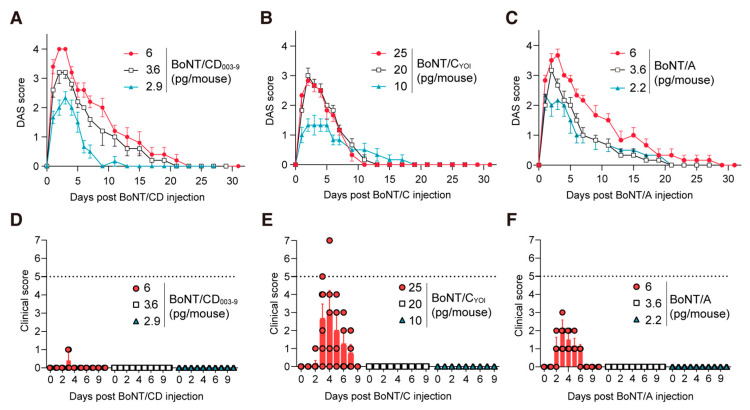
BoNT/CD_003-9_ induces local muscle paralysis more efficiently than BoNT/C_YOI_ in mice. (**A**–**C**) Local muscle paralysis model obtained by the digit abduction score assay. The indicated amount of BoNT/CD_003-9_, BoNT/C_YOI_ or BoNT/A was injected into the mouse’s hind limb muscle. The degree of muscle paralysis was scored from 0 (no paralysis) to 4 (maximum paralysis) and monitored over time. (**D**–**F**) The clinical scores of each mouse in panels (**A**–**C**) are plotted. The humane endpoint was set as a total score above 5 (dotted line). Two out of six mice that were injected with BoNT/C_YOI_ (25 pg) reached a score above 5 and were euthanized, as shown in (**E**). The data are means ± S.E. (*n* = 4–6 per dose of the toxin).

**Figure 5:**
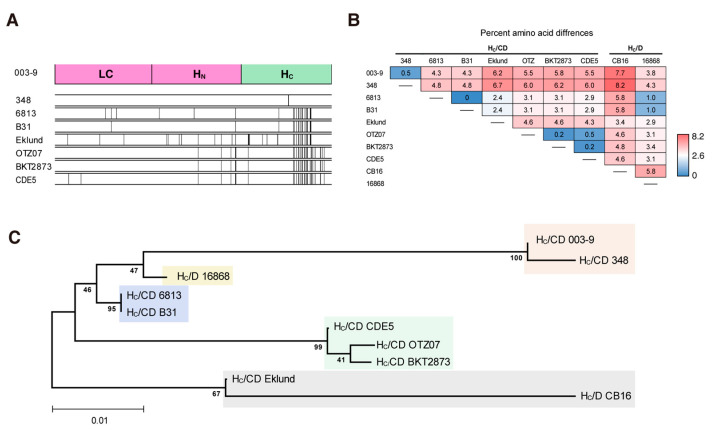
H_C_ from BoNT/CD displayed higher sequence diversity compared with LC-H_N_. (**A**) Amino acid alignments of BoNT/CDs. The differences in the amino acids of each strain are shown as black vertical lines compared with those of the 003-9 strain. (**B**) Differences in the amino acid sequences of the H_C_ of BoNT/D and BoNT/CD. Percentages are shown in shades of blue to red, which represent lower to higher differences in H_C_. (**C**) A phylogenic tree of the amino acids sequences alignment for H_C_ of BoNT/CD and BoNT/D was analyzed using the ClustalW method. Branch lengths show the evolutionary distance.

To evaluate the half-life of the BoNTs, we continuously monitored the DAS scores of the mice until paralysis in the leg disappeared (Figure 4A–C). The diffusion of intramuscular (IM)-administrated BoNTs often causes systemic paralysis, the loss of body weight, and the appearance of other typical botulism phenotypes [38]. Thus, clinical signs were also monitored to assess the systemic toxicity caused by diffused toxins (Figure 4D–F, Figure S1 and Table S1). Six picograms of BoNT/CD_003-9_ induced a DAS score of 3–4 at 2–3 days post-toxin injection without any obvious adverse clinical symptoms (Figure 4A,D). The paralysis induced by the injection of BoNT/CD_003-9_ (6 and 3.6 pg) lasted ~21 days post-injection (Figure 5A). Increasing doses of up to 8 pg BoNT/CD_003-9_ induced systemic symptoms, and five out of seven mice reached a humane endpoint (Figure S2A–C). Meanwhile, BoNT/C could not induce effective local paralysis. Twenty picograms of BoNT/C induced only a DAS score of 3 with no clinical symptoms at 2 days post-injection (Figure 4B). The mice administrated with 25 pg BoNT/C showed a DAS score of ~3, along with severe clinical symptoms, and two out of six mice were euthanized once the humane endpoint was reached (Figure 4E). The paralysis induced by BoNT/C (20 and 10 pg) lasted 13~19 days post-injection. We further evaluated the efficacy of BoNT/C using another serotype C strain (strain Stockholm; BoNT/C_STO_). BoNT/C_STO_ showed comparable efficacy with BoNT/C from the Yoichi strain (Figure S3). According to a previous report [39], BoNT/A exerts muscle paralysis over the longest period (30–40 days) in mice among the BoNT serotypes. BoNT/A (6 pg) induced maximum paralysis, with a DAS score of 3–4 at 3 days post-injection, and the paralysis lasted a total of 32 days (Figure 4C). However, these mice also showed symptoms specific to botulism, namely wasp-shaped waists, ruffled fur, and/or loss of body weight (Figure 4F). Increasing doses of up to 6.5 pg BoNT/A caused more intense systemic symptoms, and three out of six mice reached a humane endpoint (Figure S2D–F). BoNT/A (3.6 or 2.2 pg) induced a DAS score of 2–3 after IM administration, with the paralysis lasting 21 days.

We monitored the clinical scores and body weights of mice during the DAS assay. This allowed us to assess the ease of toxin diffusion to the body from the injection site. Six pg of BoNT/CD_003-9_ induced maximum paralysis (DAS score 3–4) without obvious adverse effects on the clinical score (Figure 3 and Figure 4). BoNT/C (25–30 pg/mouse), however, did not induce a DAS score of 4, although it caused systemic botulism symptoms (Figure 3 and Figure 4B,D). Donald et al. reported that BoNT/C (25 pg) induced a DAS score of 3 in mice, as well as weight loss (approximately 10%) [7]. Taken together with these data, BoNT/CD is more likely to induce local muscle paralysis compared with BoNT/C.

### 2.4. Sequence Variations in the Receptor Binding Domains of BoNT/CD

Currently, there are eight known unique protein sequences of BoNT/CD (BoNTbase.org). The BoNT/CD with the highest amino acid sequence similarity with BoNT/CD_003-9_ was from strain 348 (99.8%), and the BoNT/CD with the lowest similarity was from strain Eklund (97.0%). As shown in Figure 5A, most substitutions of amino acids were located in the H_C_. The H_C_ domain from BoNT/CD_003-9_ (H_C_/CD_003-9_) has 91% similarity with the H_C_ from BoNT/D_CB16_ (H_C_/D_CB16_; Figure 1B). We then performed an alignment analysis of the H_C_/CD and H_C_/D (eight and two stains, respectively). As shown in Figure 5B, the amino acid sequences of the H_C_/CD show differences varying within the 0–6.7% range. Interestingly, the H_C_/D strains, CB16 and 16868, also displayed relatively high differences (5.8%) in their amino acid sequences. The H_C_/D_16868_ domain displayed differences of 3.8 and 4.3% from those from the strains 003-9 and 348, respectively; these differences were lower than that between the H_C_/Ds from 16868 and CB16. On the other hand, the H_C_/D_CB16_ domain displayed the highest differences (7.7 and 8.2%) relative to those from strains 003-9 and 348, respectively. We found that there were several substitutions in the amino acid for interactions with phosphatidylethanolamine (PE) [40,41] in H_C_/CD and H_C_/D (Figure S4). K1136 in H_C_/CD_003-9_ and H_C_/CD_348_, which is responsible for interacting with PE, is replaced by Gly in other H_C_/CDs and H_C_/Ds.

We further plotted a dendrogram showing the diversity of the H_C_ domains among eight BoNT/CDs and two BoNT/Ds (Figure 5C). The results indicated that the H_C_ domains of BoNT/CD and BoNT/D could be divided into at least five groups. H_C_/CD_6813_ was classified other groups with 4.3% differences from H_C_/CD_003-9_.

### 2.5. Efficacy of BoNT/CD from Strain 6813 Compared with That from Strain 003-9

**Figure 6:**
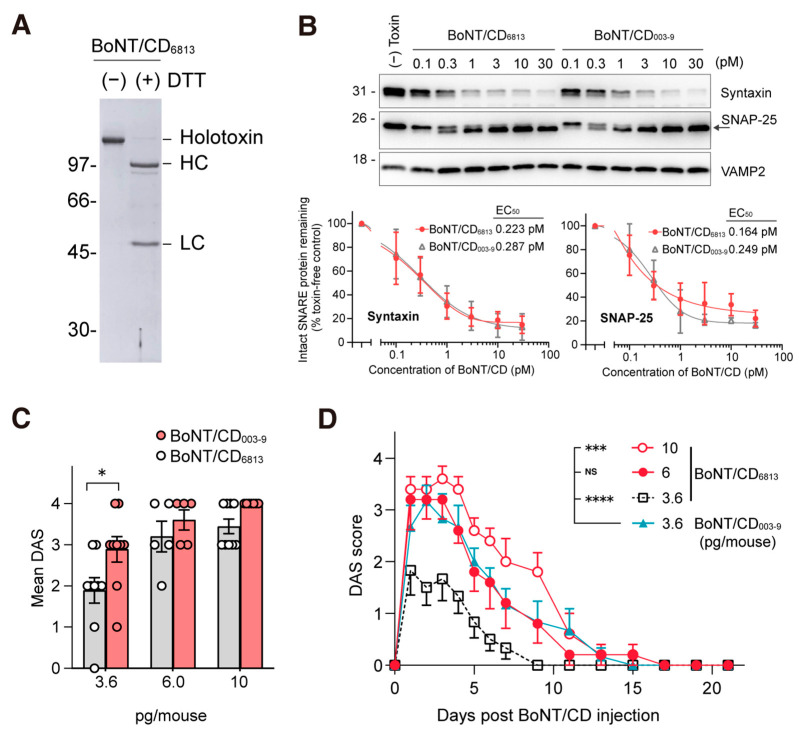
The activity of BoNT/CD_003-9_ and BoNT/CD_6813_ in vitro and in vivo in mice. (**A**) Purified BoNT/CD_6813_ from the culture supernatant of *C. botulinum*. BoNTs (1.5 µg) were analyzed on SDS-PAGE without or with DTT. (**B**) Comparison of the activity of BoNT/CD_6813_ and BoNT/CD_003-9_ in rat-cultured cortical neurons. Neurons were exposed to BoNT/CD_6813_ for 12 h. The lysate was analyzed by Western blot, and the cleavage of syntaxin and SNAP-25 was quantified. The data are means ± S.E. of three independent biological replicates. (**C**) BoNT/CD was injected into the mouse’s hind limb muscle, and muscle paralysis was scored after 3 days. The data are means ± S.E. (*n* = 6 per dose). (**D**) A comparison of the efficacy of BoNT/CD_6813_ with BoNT/CD_003-9_ in mice. Data were analyzed by two-way ANOVA with Dunnett’s multiple comparison tests, **** *p* < 0.0001, *** *p* < 0.001, * *p* < 0.1, NS, not significant.

To evaluate whether the mutations among various H_C_/CDs affect the potency of BoNT/CD, we purified BoNT/CD from the culture supernatant of strain 6813 *C. botulinum* (BoNT/CD_6813_). During SDS-PAGE analyses with reducing reagents, BoNT/CD_6813_ developed as two bands corresponding to LC and HC, indicating that the BoNT exists as an active form in culture without exogenous protease treatment (Figure 6A). In the cultured rat cortical neuron assay, BoNT/CD_6813_ cleaved syntaxin and SNAP-25 with almost identical efficacy, as did BoNT/CD_003-9_ (Figure 6B). In the mouse DAS assay, 3.6 pg of BoNT/CD_6813_ induced a DAS score of only 2 at 3 days post-administration; meanwhile, equivalent doses of BoNT/CD_003-9_ induced a DAS score of 3 (Figure 6C). As shown in Figure 6D, the DAS score and duration of paralysis induced by 3.6 pg of BoNT/CD_003-9_ were similar to those induced by 6 pg of BoNT/CD_6813_. Moreover, the administration of 10 pg of BoNT/CD_6813_ in mice induced a DAS score of 3–4 without adverse clinical symptoms (Figure 6D and Figure S5).

## 3. Discussion

The range of applications of BoNT in medical and cosmetic fields is rapidly expanding [3,42,43]. In some patients, the immune response from the repeated administration of toxins may diminish therapeutic efficacies [7,44,45,46]. In this study, we characterized and compared the efficacy of BoNT/CD and long-lasting BoNT/C and BoNT/A. Our results demonstrate that BoNT/CD efficiently induces local muscle paralysis compared with the BoNT/C parental toxin. BoNT/CD showed substantially more activity in cultured rat cortical neurons compared with BoNT/A and BoNT/C. Muscle paralysis was induced by BoNT/CD_003-9_ for 21 days in our local injection mouse model. Furthermore, the H_C_/D of BoNT/CD and BoNT/D can be classified into at least five groups based on amino acid sequences. Our study demonstrated that BoNT/CD offers promise as an alternative to BoNT/A in inducing local muscle paralysis for the treatment of muscle disorders. 

BoNT/CD (both strains 003-9 and 6813), BoNT/C, and BoNT/A purified from their native *C. botulinum* strains are naturally activated without exogenous protease treatments (Figure 1B). Non-activated BoNTs, especially BoNT/E, require additional protease treatment. This may make it difficult to obtain uniform toxins between batches, and excess proteolysis may also induce unwanted cleavage [47]. The specific protease responsible for the activation of BoNT/CD is currently unknown; however, the BoNT/CDs employed in this study are available as fully activated toxins without the need for additional proteolytic activation processes.

The activity of BoNT/CD_003-9_ was investigated in cultured neurons. BoNT/CD_003-9_ displayed a potency that was 35.1 times higher than that of BoNT/C_YOI_ (Figure 2C). Furthermore, the potency of BoNT/CD_003-9_ was higher than that of BoNT/A by 4.3 times. In contrast, BoNT/A showed higher efficacies in an animal model compared with BoNT/CD_003-9_ (Figure 3). This discrepancy may be related to the expression of receptors on motor neurons in vivo and cultured cortical neurons, and it may depend on the differences in animal species’ susceptibility. These results coincide with that of mosaic toxin BoNT/FA; its efficacy is higher than that of BoNT/A in cultured neurons but not in mice [48]. BoNT/CD_003-9_ induced a shorter half-life with respect to muscle paralysis than BoNT/A (21 and 30 days, respectively). The local muscle paralysis model also suggests that BoNT/CD_003-9_ can cause local paralysis with less systemic diffusion than BoNT/A while exhibiting efficacy comparable to that of BoNT/A (Figure 4A,B). Since BoNT/CD_003-9_ and BoNT/A cause intense systemic toxicity by increasing the doses up to 8 pg or 6.5 pg, respectively (Figure S2), these toxins are expected to have a similar safety range. Further studies are needed to clarify the safety or therapeutic window of the BoNT/CD.

We found that BoNT/CD can be categorized into at least five groups. BoNT/CD_003-9_ was also found to be more potent than BoNT/CD_6813_ (Figure 6C,D). These data suggest that the differences in the H_C_ domain of BoNT/CD may affect in vivo toxicity. The H_C_ of BoNT/D has dual polysialioganglioside (PSG) binding sites [49]. Two PSG binding sites are conserved in all BoNT/CDs (Figure S4). Additionally, BoNT/D may utilize SV2 as a protein receptor, although the binding mechanism and responsible amino acid residues have not yet been fully elucidated [28], rendering it difficult to assess the contribution of residue variations within the H_C_. 

BoNT/C and BoNT/D rarely cause human botulism [1]. These toxins, however, have undergone preclinical testing for their application in therapeutics [14]. BoNT/C is recognized as a second-rank toxin with long half-life muscle paralysis, similar to that of BoNT/A [14,16]. We found that BoNT/C is less potent than BoNT/CD_003-9_ in mice. Increasing a dose of BoNT/C caused systemic toxicity and failed to induce complete paralysis in local muscles (Figure 4E and Figure S2C); however, BoNT/CD has similar potencies with BoNT/A. In contrast, BoNT/D (strain CB16) is less efficacious in humans; nevertheless, it targets the SV2 protein, such as a BoNT/A [17], as there is a mutation in the cleavage site of VAMP, which is a substrate for LC/D [25], although increasing injection dosages still cause paralysis. 

## 4. Conclusions

The current study reveals the potency of a mosaic botulinum neurotoxin, BoNT/CD, comprising serotype C LC-H_N_ and serotype D H_C_ domains in cultured rat cortical neurons and a model for local muscle paralysis in mice. BoNT/CD had a higher efficacy in neuron assay compared to BoNT/C and BoNT/A. BoNT/CD, however, induced similar efficacy in the muscle paralysis model with less systemic toxicity compared to BoNT/A in mice. BoNT/CD is more effective than BoNT/C in inducing local muscle paralysis, and the result indicates that H_C_ has a significant influence on the efficacy of the toxicity. 

## 5. Materials and Methods

### 5.1. Materials

Mouse monoclonal antibodies for SNAP-25 (SP-12, diluted 1:2000) and syntaxin 1 (HPC-1, 1:2000) were purchased from Santa Cruz Biotechnology (Dallas, TX, USA). Rabbit monoclonal anti-VAMP 2 antibodies (D6O1A, 1:1000) were purchased from Cell Signaling Technology (Danvers, MA, USA). Secondary antibodies conjugated with horse radish peroxidase (1:5000) were purchased from Cell Signaling Technology.

### 5.2. Production and Purification of Neurotoxins 

BoNTs were produced from *C. botulinum* by using dialysis-tube methods as previously described [50,51]. *C. botulinum* serotype CD (strains 003-9 and 6813), serotype C (strain Yoichi and Stockholm), and serotype A (strain 62A) were used to produce the botulinum neurotoxins (BoNT/CD strain 003-9; GenBank: BAD90568.1, BoNT/CD strain 6813; GenBank: BAA08418.1, BoNT/C strain Yoichi; GenBank: BAB71749.1, BoNT/C strain Stockholm; GenBank: P18640.3, BoNT/A strain 62A; GenBank: ACS52162.1, respectively). L-PTC fractions were dialyzed with 50 mM acetate buffer (pH 5.0) at 4 °C overnight. L-PTCs were purified on Macro-Prep High S (Bio-Rad laboratories, Hercules, CA, USA). L-PTCs were collected based on the molecular weight as obtained using SDS-PAGE analysis. To isolate the BoNT from the L-PTC, L-PTC was dialyzed with 20 mM Tris-HCl (pH 8.8) and 400 mM NaCl at 4 °C overnight and concentrated using Amicon Ultra (100-kDa MWCO, Millipore). Proteins were loaded onto a Superdex 200 10/300 GL (GE Healthcare). The BoNT peak was collected and diluted eight-fold with 20 mM Tris-HCl (pH 8.0). The BoNTs were further purified by a linear gradient of 0 to 500 mM NaCl over 20 mL using Mono Q 5/50 GL (GE Healthcare, Chicago, IL, USA). BoNTs were concentrated using Amicon Ultra-0.5 (50-kDa MWCO, Millipore, Burlington, MA, USA) and sterilized using Ultrafree-MC Centrifugal Filter (0.22-µm, GV Durapore, Millipore). The protein concentrations were quantified by absorbance at 280 nm or followed the instructions of Pierce BCA Proteins Assay Kit (Thermo Fisher Scientific, Waltham, MA, USA). Purified BoNTs were stored at 4 °C for several months. 

### 5.3. Neuron Culture

Primary embryonic cortical neurons from pregnant rats (embryonic day 19) were prepared as previously described [38]. Briefly, the cortex of the brain was dissected from the embryo and purified by a papain dissection kit (Worthington Biochemical, Lakewood, NJ, USA). Twenty-four-well plates were coated with 0.1 mg/mL poly-D-lysine (PDL) for 3 h and washed with MilliQ water. Cortical neurons were plated on the PDL-coated plates at a density of 200,000 cells per well in 1 mL of B-27 Plus Neuronal Culture System (Thermo Fisher Scientific; #A3653401) containing 2 mM GlutaMAX (Thermo Fisher Scientific) and 1% antibiotic antimycotic solution (Sigma-Aldrich, St. Louis, MO, USA). DIV 14-30 neurons were used for the experiments.

### 5.4. SNARE Protein Cleavage in Cultured Neurons

The cleavage of SNARE proteins by BoNTs on cultured neurons was detected as previously described [38]. Neurons were exposed to BoNT/CD, BoNT/C, or BoNT/A in 400 µL of culture medium for 12 h at 37 °C. Cells were washed with PBS once and lysed in 200 µL of lysis buffer [PBS containing 1% Triton X-100, 0.05% SDS, and protease inhibitor cocktail (Nacalai Tesque)]. The lysate was centrifuged at maximum speed for 10 min at 4 °C. The supernatants were stored at −80 °C until use and mixed with SDS sample buffer [62.5 mM Tris-HCl (pH 6.8), 2% SDS, 7.5% glycerol, and 0.005% bromophenol blue]. Proteins were separated on a 12.5% SDS-polyacrylamide gel and transferred onto a nitrocellulose membrane (Cytiva). Membranes were incubated in blocking buffer [TBS-T; 5 mM Tris-HCl (pH 7.4), 13.8 mM NaCl, 0.27 mM KCl, and 0.05% Tween 20, containing 5% (*w/v*) non-fatty milk] for 1 h at room temperature. Primary antibodies were incubated overnight at 4 °C in a blocking buffer. Membranes were washed with TBS-T three times and incubated with secondary antibodies in a blocking buffer for 1 h. Membranes were washed with TBS-T 3 times, and the proteins were imaged with Light-Capture II (ATTO, Tokyo, Japan). Original images of the Western blot were shown in Figure S6.

### 5.5. Sequence Analysis

The amino acid sequences of 8 strains of BoNT/CD and 2 strains of BoNT/D were analyzed. MEGA11 was used for multiple alignments and the construction of the molecular phylogenetic. The tree with the highest log likelihood (−1480.75) is shown. The percentage of trees in which the associated taxa clustered together is shown next to the branches. Initial tree(s) for the heuristic search were obtained automatically by applying Neighbor-Join and BioNJ algorithms to a matrix of pairwise distances estimated using the JTT model and then selecting the topology with superior log likelihood value. A discrete Gamma distribution was used to model evolutionary rate differences among sites (5 categories (+G, parameter = 0.0692)). This analysis involved 10 amino acid sequences. There was a total of 417 positions in the final dataset. Evolutionary analyses were conducted in MEGA11.

### 5.6. DAS Assay 

Toxicity of BoNT in mouse local muscle was assessed as previously reported [37,38]. Female Crl: CD1(ICR) mice were purchased from Charles River Japan. Mice weighing 20–30 g were anesthetized with isoflurane and injected with BoNTs in 10 µL of 0.2% gelatin-phosphate buffer (pH 6.3) in the right hind limb muscle. Mice were suspended by the tail briefly, and the degree of digit abduction of the leg was scored on a scale of 0 (normal) to 4 (maximum paralysis). The paralysis scores, body weights, and clinical scores (Table S1) of the mice were monitored at least two times per day for 4 d, once per day for 7 d, and then once every other day. The humane endpoint was set as a total clinical score of above 5.

### 5.7. Statistical Analysis

Statistical analyses were performed with GraphPad Prism 9, and significance was determined by performing a two-way ANOVA with Dunnett’s multiple comparisons test. All in vitro experiments were performed in triplicate unless otherwise indicated. All in vivo experiments were performed in at least duplicate with similar conditions unless otherwise indicated. 

### 5.8. Ethics Statement

All animal experiments were performed in accordance with the Guidelines for Proper Conduct of Animal Experiments as promulgated by the Science Council of Japan. The study design was reviewed and approved by the Animal Care and Use Committee of Tokyo University of Agriculture (protocol number 2020074).

## Data Availability

The methods and data and supplementary data presented in this study are available.

## Supplementary Materials

The following supporting information can be downloaded at: https://www.mdpi.com/article/10.3390/toxins15020123/s1, Figure S1: Effect of body weight by the intramuscular injection of BoNT; Figure S2: Intramuscular injection of BoNT/CD_003-9_ (8 pg) and BoNT/A (6.5 pg) induced the loss of body weight and systemic toxicity; Figure S3: Efficacy of BoNT/C Stockholm as observed with a DAS assay; Figure S4: Alignment of amino acid sequences of the receptor binding domains of BoNT/CD and BoNT/D; Figure S5: Systemic toxicity of BoNT/CD_6813_ in DAS assays. The clinical scores of each mouse in Figure 6D are plotted; Figure S6: Original images of the Western blot; Table S1: Clinical Scores for botulism in mice.
