# Supplementary material for: Characterization of Serotype CD Mosaic Botulinum Neurotoxin in Comparison with Serotype C and A
Source: Toxins (Basel). 2023 Feb 3;15(2):123. doi: 10.3390/toxins15020123 (PMC9962336; doi:10.3390/toxins15020123)
Supplement: Supplementary file 1 [file toxins-15-00123-s001.zip › toxins-2134476-supplementary.pdf]

Supplementary Materials: Characterization of Serotype CD Mosaic Botulinum Neurotoxin in Comparison with Serotype C and A

Shin-Ichiro Miyashita, Shura Karatsu, Mako Fujiishi, I Hsun Huang, Yuki Nagashima, Tamaki Morobishi, Keita Hosoya, Tsuyoshi Hata, Min Dong and Yoshimasa Sagane

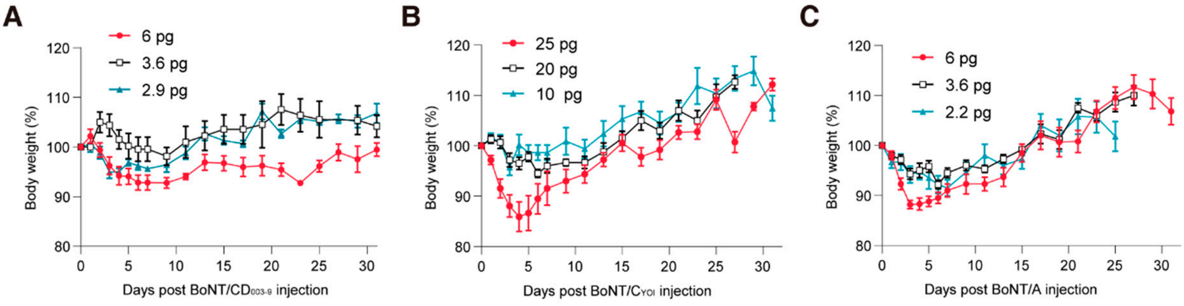

Figure S1. Effect of body weight by the intramuscular injection of BoNT. Body weights were monitored every 1–2 days, as shown in Figure 5: BoNT/CD$_{003\text{-}9}$ (**A**), BoNT/C$_{\text{YOI}}$ (**B**), and BoNT/A (**C**). The data are means ± S.E. (n = 4–6 per dose of the toxin).

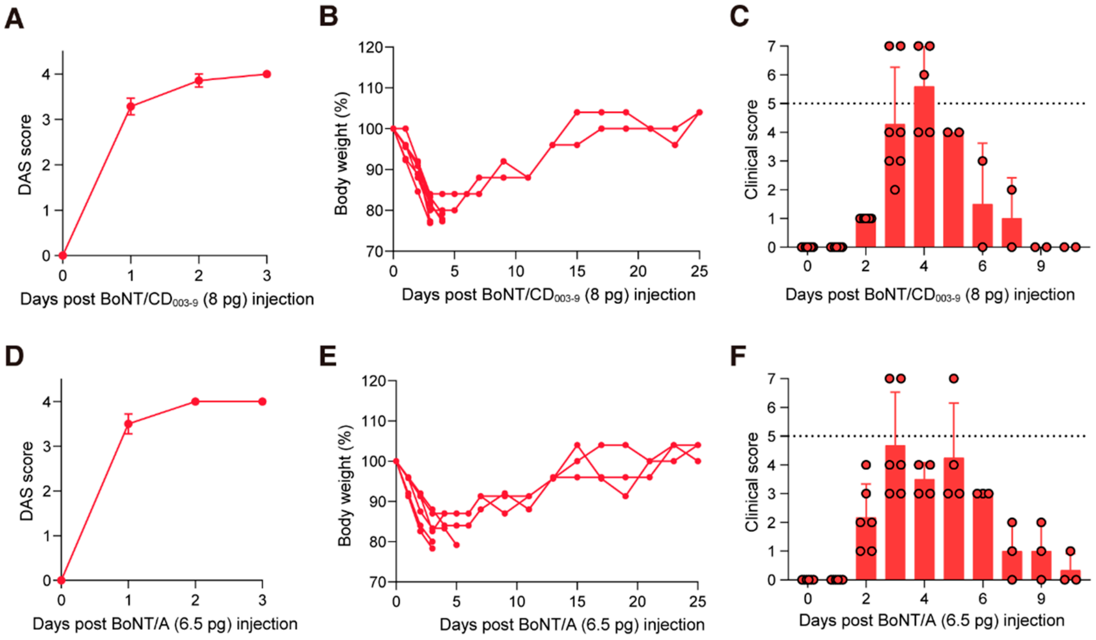

Figure S2. Intramuscular injection of BoNT/CD$_{003\text{-}9}$ (8 pg) and BoNT/A (6.5 pg) induced the loss of body weight and systemic toxicity. BoNT/CD$_{003\text{-}9}$ at 8 pg or BoNT/A at 6.5 pg was injected into the mouse hind limb muscle (n = 7, 6 respectively). The extent of muscle paralysis (**A**,**D**), body weight (**B**,**E**), and clinical score (**C**,**F**) were monitored over time. Five out of seven mice in BoNT/CD$_{003\text{-}9}$ and three out of six mice in BoNT/A reached a humane endpoint (score above 5) and were euthanized. The data are means ± S.D.

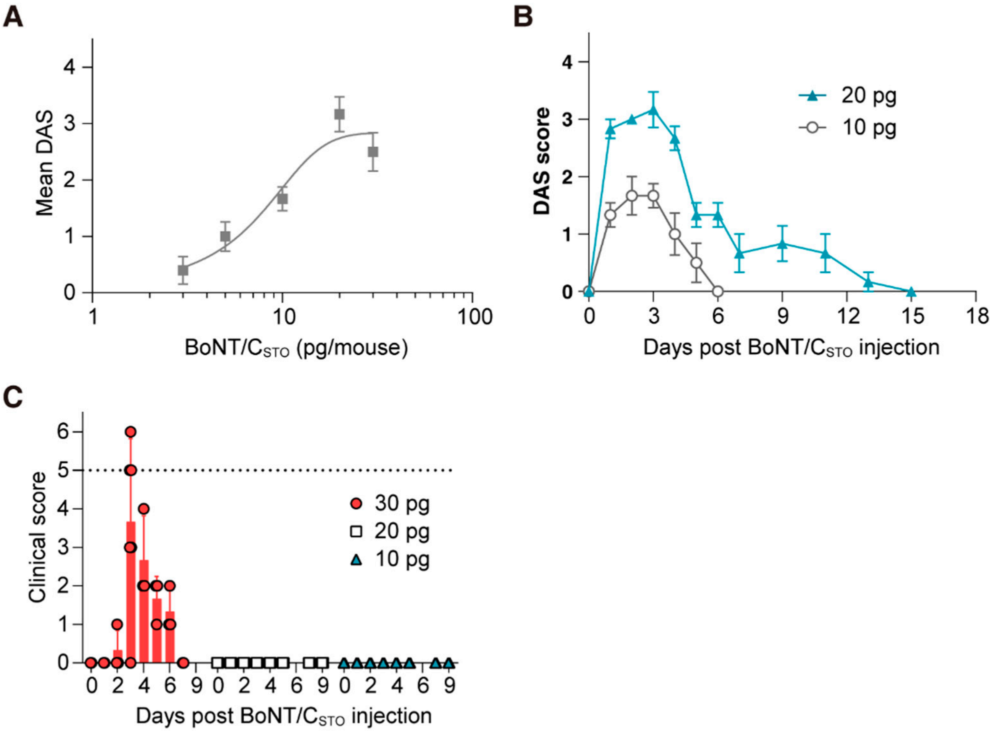

Figure S3. Efficacy of BoNT/C Stockholm as observed with a DAS assay. The indicated amount of BoNT/C Stockholm (BoNT/C_{sto}) was injected into the mouse's hind limb muscle. The degree of muscle paralysis after 3 days (**A**), the duration of paralysis (**B**), and clinical score (**C**) were monitored over time.

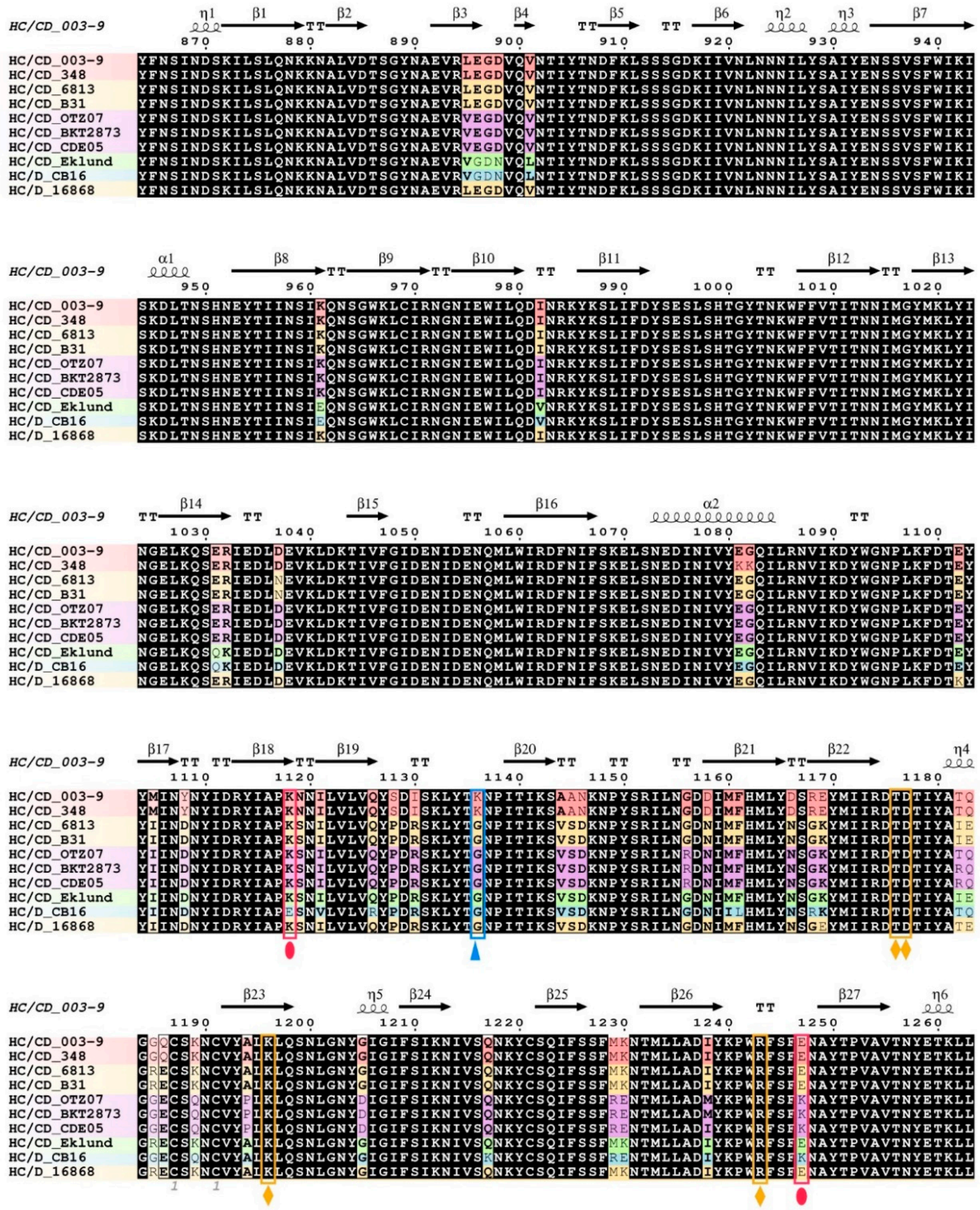

Figure S4. Alignment of amino acid sequences of the receptor binding domains (H$_C$) of BoNT/CD and BoNT/D. Alignment was performed using ClustalW in MEGA11, and displayed using ESPript (https://espript.ibcp.fr).HC/D in BoNT/D interacts with polysialoganglioside (PSG) and phosphatidylethanolamine (PE) [49]. The sialic acid binding sites of HC/D comprising Thr1172, Asp1173, Lys1192, and Arg1239 are conserved in all HC/CDs as Thr1176, Asp1177, Lys1196, and Arg1243, respectively. Yellow rhombuses indicate the location of the sialic acid binding site). K1118 and K1136 in HC/CD$_{003\text{-}9}$ are responsible for binding to PE and the synaptosome [41]. K1118 is conserved in all HC/CDs and HC /D$_{16868}$ as K1114, but not in HC/DCB16, in which the Lys is replaced by E1114. K1136 is conserved in two BoNT/CDs from strains 003-9 and 348 but not in other BoNT/CDs in which Lys is replaced by Gly. Red circles indicate K1118 and E1247, which are involved in the interactions with PE and the synaptosome. Blue triangles indicate the K1136 of H$_C$/CD$_{003\text{-}9}$ and H$_C$/CD$_{348}$.

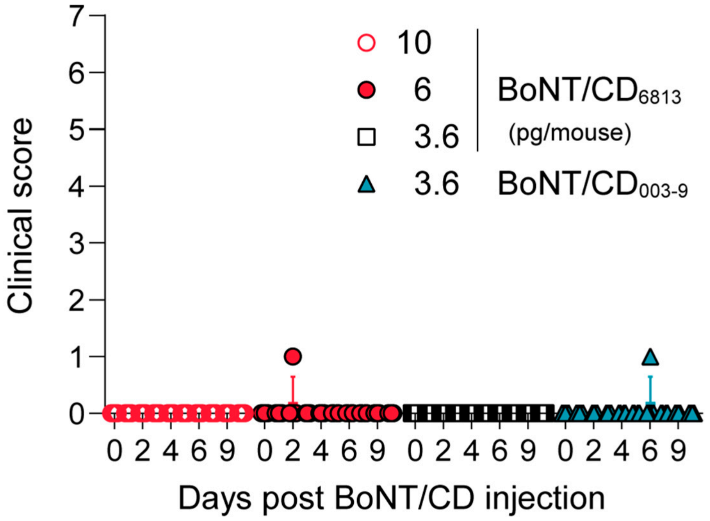

Figure S5. Systemic toxicity of BoNT/CD_{6813} in DAS assays. The clinical scores of each mouse in Figure 6D are plotted.

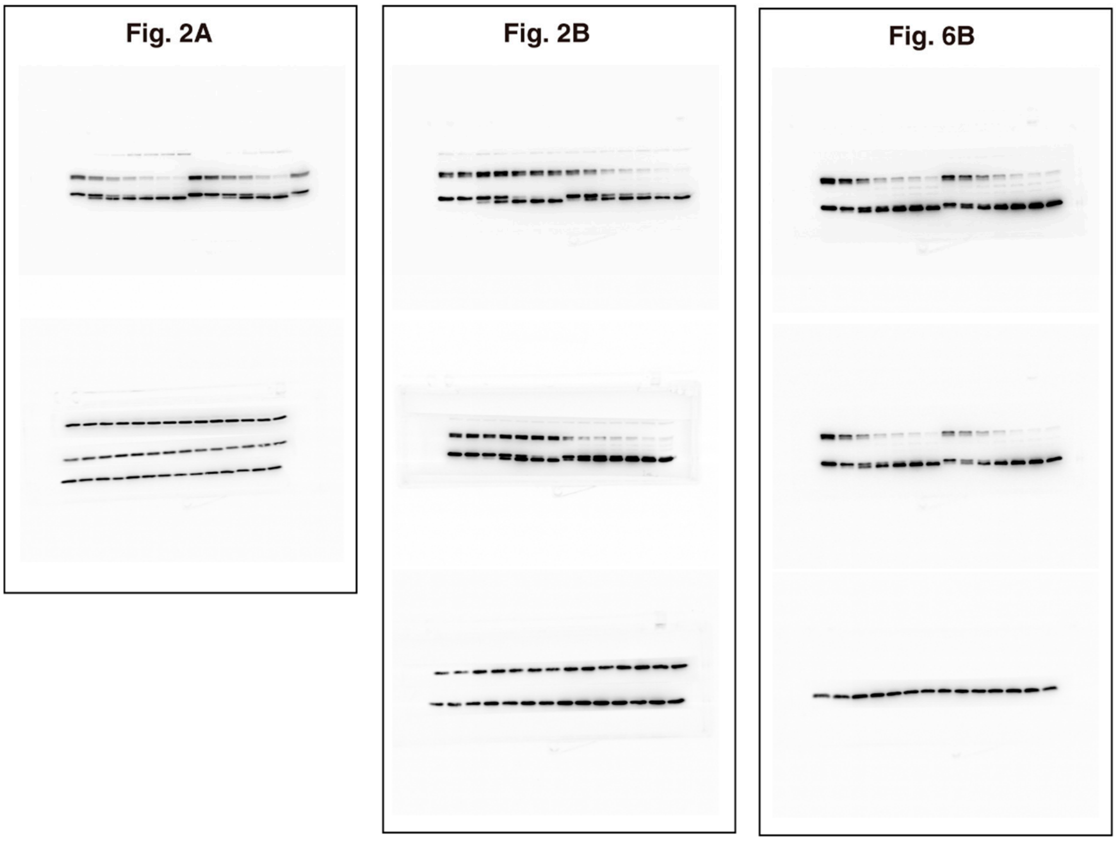

Figure S6. Original images of the Western blot.

Table S1. Clinical scores for botulism in mice.

Parameter	Degree	Score
Fur	Shining	0
	Ruffled	1
Posture	Normal	0
	Wasp waist	1
Motility	Spontaneous	0
	Spontaneous but reduced	1
	Moderately reduced	2
	Motility only after stimulation	3
	Isolation, paralysis	5
Respiration	Normal	0
	Breathing slightly changed	3
	Open mouse shallow (throat)	5
	Gasping and jerky (abdominal)	5
Bodyweight	Loss of body weight (>10%)	1
	Loss of body weight (>15%)	2
	Loss of body weight (>20%)	5
